# Supplementary material for: Nutritional Interventions in Pancreatic Cancer: A Systematic Review
Source: Cancers (Basel). 2022 Apr 28;14(9):2212. doi: 10.3390/cancers14092212 (PMC9101959; doi:10.3390/cancers14092212)
Supplement: Supplementary file 1 [file cancers-14-02212-s001.zip › cancers-1680438-supplementary.pdf]

Supplementary Materials

Nutritional Interventions in Pancreatic Cancer: A Systematic Review

Aline Emanuel, Julia Krampitz, Friederike Rosenberger, Sabine Kind and Ingeborg Rötzer

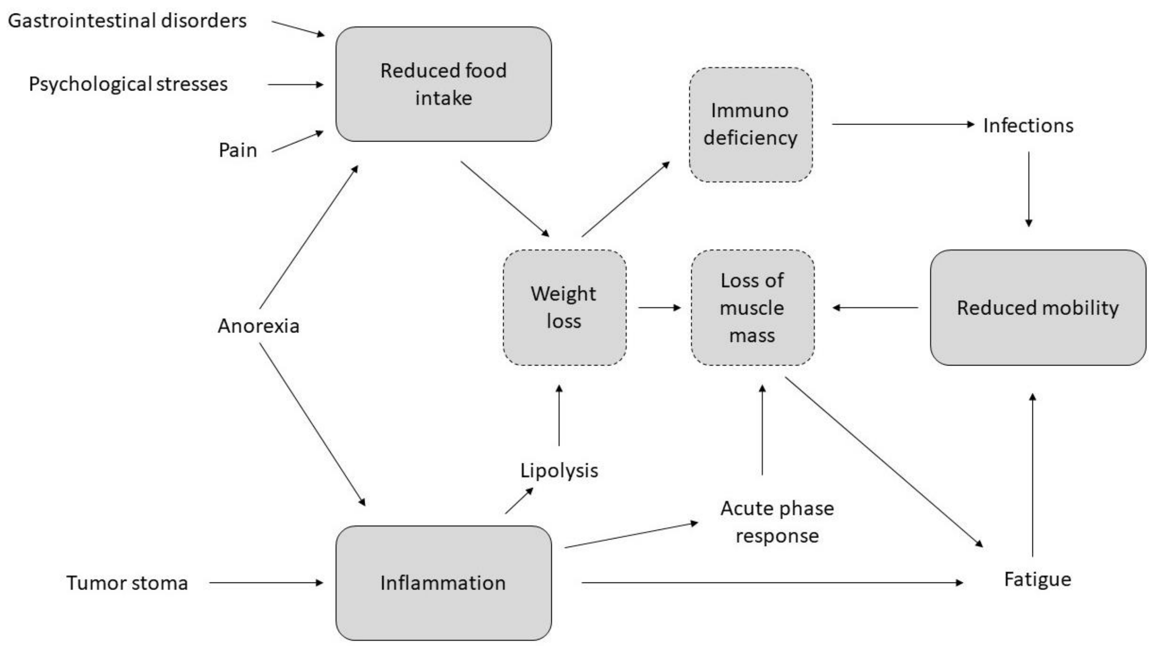

Figure S1: Tumour cachexia by Arends et al. 2012.
